# Maximum Likelihood and Restricted Likelihood Solutions in Multiple-Method Studies

**DOI:** 10.6028/jres.116.004

**Published:** 2011-02-01

**Authors:** Andrew L. Rukhin

**Affiliations:** National Institute of Standards and Technology, Gaithersburg, MD 20899-8980

**Keywords:** DerSimonian-Laird estimator, Groebner basis, heteroscedasticity, interlaboratory studies, iteration scheme, Mandel-Paule algorithm, meta-analysis, parametrized solutions, polynomial equations, random effects model

## Abstract

A formulation of the problem of combining data from several sources is discussed in terms of random effects models. The unknown measurement precision is assumed not to be the same for all methods. We investigate maximum likelihood solutions in this model. By representing the likelihood equations as simultaneous polynomial equations, the exact form of the Groebner basis for their stationary points is derived when there are two methods. A parametrization of these solutions which allows their comparison is suggested. A numerical method for solving likelihood equations is outlined, and an alternative to the maximum likelihood method, the restricted maximum likelihood, is studied. In the situation when methods variances are considered to be known an upper bound on the between-method variance is obtained. The relationship between likelihood equations and moment-type equations is also discussed.

## 1. Introduction: Meta Analysis and Interlaboratory Studies

This article is concerned with mathematical aspects of an ubiquitous problem in applied statistics: how to combine measurement data nominally on the same property by methods, instruments, studies, medical centers, clusters or laboratories of different precision. One of the first approaches to this problem was suggested by Cochran [1], who investigated maximum likelihood (ML) estimates for one-way, unbalanced, heteroscedastic random-effects model. Cochran returned to this problem throughout his career [2, 3], and Ref. [4] reviews this work. Reference [5] discusses applications in metrology and gives more references.

The problem of combining data from several sources is central to the broad field of meta-analysis. It is most difficult when the unknown measurement precision varies among methods whose summary results may not seem to conform to the same measured property. Reference [6] provides some practical suggestions for dealing with the problem.

In this paper we investigate the ML solutions of the random effects model which is formulated in Sec. 2. By representing the likelihood equations as simultaneous polynomial equations, the so-called Groebner basis for them is derived when there are two sources. A parametrization of such solutions is suggested in Sec. 2.1. The maxima of the likelihood function are compared for positive and zero between-labs variance. A numerical method for solving likelihood equations by reducing them to an optimization problem for a homogeneous objective function is given in Sec. 2.2. An alternative to the ML method, the restricted maxi mum likelihood is considered in Sec. 3. An explicit formula for the restricted likelihood estimator is discovered in Sec. 3.2 in the case of two methods. Section 4 deals with the situation when methods variances are considered to be known, and an upper bound on the between-method variance is obtained. The Sec. 5 discusses the relationship between likelihood equations and moment-type equations, and Sec. 6 gives some conclusions. All auxiliary material related to an optimization problem and to elementary symmetric polynomials is collected in the Appendix.

## 2. ML Method and Polynomial Equations

To model interlaboratory testing situation, denote by *n_i_* the number of observations made in laboratory *i*, *i* = 1, …, *p*, whose observations *x_ij_* have the form

(1) $$x_{ij}=\mu +b_{i}+\varepsilon_{ij}.$$

Here *μ* is the true property value, *b_i_* represents the method (or laboratory) effect, which is assumed to be normal with mean 0 and unknown variance *σ*^2^, and *ε_ij_* represent independent normal, zero mean random errors with unknown variances $\tau_{i}^{2}$.

For a fixed *i*, the *i*-th sample mean *x_i_* = Σ*_j_ x_ij_*/*n_i_* is normally distributed with the mean *μ* and the variance *σ*^2^ + *σ_i_*^2^, where ${\sigma_{i}}^{2}=\tau_{i}^{2}/n_{i}$. If the *σ*’s were known, then the best estimator of *μ* would be the weighted average of *x_i_* with weights proportional to 1/(*σ*^2^+ *σ_i_*^2^). Since these variances are unknown, the weights have to be estimated. Traditionally to evaluate *σ_i_*^2^ one uses the classical unbiased statistic *s_i_*^2^ = Σ*_j_*(*x_ij_*−*x_i_*)^2^/(*n_i_v_i_*), *v_i_* = *n_i_*−1, which has the distribution *σ_i_*^2^*χ*^2^(*v_i_*)/*v_i_*. Since statistics *x_i_*, *s_i_*^2^, *i* = 1, …, *p*, are sufficient, we use the likelihood function based on them.

The ML solution minimizes in *μ, σ*^2^, *σ_i_*^2^, *i* = 1, …, *p*, the negative logarithm of this function which is proportional to

(2) $$\begin{array}{l} L=\sum_{i}\frac{{\left(x_{i}-\mu \right)}^{2}}{\sigma^{2}+{\sigma_{i}}^{2}}+\sum_{i}\frac{v_{i}s_{i}^{2}}{{\sigma_{i}}^{2}}+\\ \;\sum_{i}\log(\sigma^{2}+{\sigma_{i}}^{2})+\sum_{i}v_{i}\log{\sigma_{i}}^{2}. \end{array}$$

It follows from (2) that if ${\hat{\sigma }}_{i}^{2}$ is the ML estimator of *σ_i_*^2^, then ${\hat{\sigma }}_{i}^{2}>0$. However, it is quite possible that ${\hat{\sigma }}_{i}^{2}=0$. In order to find these estimates one can replace *μ* in (2) by

(3) $$\bar{\mu }=\frac{\sum_{i}x_{i}{\left(\sigma^{2}+\sigma_{i}^{2}\right)}^{-1}}{\sum_{i}{\left(\sigma^{2}+\sigma_{i}^{2}\right)}^{-1}},$$

which reduces the number of parameters from *p* + 2 to *p* + 1.

Our goal is to represent the set of all stationary points of the likelihood equations as solutions to simultaneous polynomial equations. To that end, note that

(4) $$\begin{array}{c} \sum_{i}w_{i}{\left(x_{i}-\bar{\mu }\right)}^{2}=\sum_{i}w_{i}x_{i}^{2}-{\left(\sum_{i}w_{i}x_{i}^{2}\right)}^{2}/\left(\sum_{i}w_{i}\right)\\ =\sum_{1\leq i\leq j\leq p}w_{i}w_{j}{\left(x_{i}-x_{j}\right)}^{2}/\left(\sum_{i}w_{i}\right). \end{array}$$

This formula, which easily follows from the Lagrange identity [7, Sec. 1.3], will be used with *w_i_* = (*σ*^2^+ *σ_i_*^2^)^−1^.

We introduce a polynomial *P* of degree *p* in *σ*^2^,

(5) $$P=P(\sigma^{2},\sigma_{1}^{2},\ldots ,\sigma_{p}^{2})=\prod_{i}\left(\sigma^{2}+\sigma_{i}^{2}\right)=\sum_{\ell =0}^{p}S_{p-\ell }\sigma^{2\ell }$$

with $S_{\ell }=S_{\ell }(\sigma_{1}^{2},\ldots ,\sigma_{p}^{2})$, *ℓ* = 0,1,…, *p*, denoting the *ℓ*-th elementary symmetric polynomial. Another polynomial of interest is $Q=Q(\sigma^{2},\sigma_{1}^{2},\ldots ,\sigma_{p}^{2})$ having degree *p*−2 in *σ*^2^,

(6) $$\begin{array}{c} Q(\sigma^{2},\sigma_{1}^{2},\ldots ,\sigma_{p}^{2})=\sum_{1\leq i\leq j\leq p}{\left(x_{i}-x_{j}\right)}^{2}\prod_{k\neq i,j}\left(\sigma^{2}+\sigma_{k}^{2}\right)\\ =\sum_{\ell =0}^{p-2}q_{p-2-\ell }\sigma^{2\ell }=\sum_{1\leq i\leq j\leq p}{\left(x_{i}-x_{j}\right)}^{2}P_{ij}, \end{array}$$

where $P_{ij}=\frac{\partial^{2}}{\partial \sigma_{1}^{2}\partial \sigma_{j}^{2}}P(\sigma^{2},\sigma_{1}^{2},\ldots ,\sigma_{p}^{2})$ is a polynomial in *σ*^2^ of degree *p*−2 which does not depend on $\sigma_{i}^{2}$, $\sigma_{j}^{2}$, and $q_{\ell }=q_{\ell }(\sigma_{1}^{2},\ldots ,\sigma_{p}^{2})$ is a multilinear form in $\sigma_{1}^{2},\ldots ,\sigma_{p}^{2}$.

Since

(7) $$\frac{P^{\prime }}{P}=\frac{\partial }{\partial \sigma^{2}}\log P(\sigma^{2},\sigma_{1}^{2},\ldots ,\sigma_{p}^{2})=\sum_{k}\frac{1}{\sigma^{2}+\sigma_{k}^{2}},$$

the identity (4) can be written as

(8) $$\sum_{k}\frac{{\left(x_{k}-\bar{\mu }\right)}^{2}}{\sigma^{2}+\sigma_{k}^{2}}=\frac{Q/P}{P^{\prime }/P}=\frac{Q}{P^{\prime }}.$$

The negative log-likelihood function (2) in this notation is

(9) $$L=\frac{Q}{P^{\prime }}+\log P+\sum_{i}\frac{v_{i}s_{i}^{2}}{\sigma_{i}^{2}}+\sum_{i}v_{i}\log\sigma_{i}^{2}.$$

Let $P_{i}=\prod_{k\neq i}\left(\sigma^{2}+\sigma_{k}^{2}\right)$be the partial derivative of *P* with regard to $\sigma_{i}^{2}$; denote by *Q_i_* the same partial derivative of *Q* and by $P_{i}^{\prime }=\sum_{j:j\neq i}P_{ij}$ the derivative of $P^{\prime }\left(\sigma^{2},\sigma^{2},\ldots ,\sigma_{p}^{2}\right)$. By differentiating (9), we see that the stationary points of (2) satisfy polynomial equations,

(10) $$\begin{array}{r} \sigma_{i}^{4}\left(\sigma^{2}+\sigma_{i}^{2}\right)\left(Q_{i}P^{\prime }-QP_{i}^{\prime }\right)+\sigma_{i}^{4}{\left(P^{\prime }\right)}^{2}\\ +v_{i}\left(\sigma_{i}^{2}-s_{i}^{2}\right)\left(\sigma^{2}+\sigma_{i}^{2}\right)P^{\prime }=0. \end{array}$$

Each of these polynomials has degree 4 in $\sigma_{i}^{2}$, *i* = 1, …, *p*. When $\sigma^{2}=0$, $P=\prod \sigma_{i}^{2}$, and the equations (10) simplify to

(11) $$\begin{array}{r} \sigma_{i}^{2}P\left(Q_{i}P^{\prime }-QP_{i}^{\prime }\right)+\sigma_{i}^{2}P_{i}{\left(P^{\prime }\right)}^{2}\\ +v_{i}\left(\sigma_{i}^{2}-s_{i}^{2}\right)P_{i}{\left(P^{\prime }\right)}^{2}=0. \end{array}$$

These polynomials have degree 3 in $\sigma_{i}^{2}$. If $\sigma^{2}>0$, in addition to (10) one has

(12) $$F={\left(P^{\prime }\right)}^{3}+\left(Q^{\prime }P^{\prime }-QP^{\prime \prime }\right)P=0,$$

and *F* has degree 3*p*−3 in *σ*^2^.

In both cases the collection of all stationary points forms an affine variety whose structure can be studied via the *Groebner basis* of the ideal of polynomials (11) or (10) and (12) which vanish on this variety. The Groebner basis allows for successive elimination of variables leading to a description of the points in the variety, i.e., to the characterization of all (complex) solutions. There are powerful numerical algorithms for evaluation of such bases [8]. Many polynomial likelihood equations are reviewed in Ref. [9]. We determine the Groebner basis for equations (11) when *p* = 2 in the next section.

### 2.1 ML Method: *p* = 2

When *p* = 2, *Q* = (*x*_1_−*x*_2_)^2^. If *σ*^2^ = 0, the polynomial equations (11) take the form

(13) $${\left(x_{1}-x_{2}\right)}^{2}\sigma_{i}^{4}-\left(n_{i}\sigma_{i}^{2}-v_{i}s_{i}^{2}\right){\left(\sigma_{1}^{2}+\sigma_{2}^{2}\right)}^{2}=0,i=1,2.$$

The Groebner basis is useful for solving these equations as it allows elimination of one of the variables, say, $\sigma_{2}^{2}$. With *n* = *n*_1_ + *n*_2_, *f* = *n* + *n*_2_, $u=\sigma_{1}^{2}/{\left(x_{1}-x_{2}\right)}^{2}$, $\upsilon =\sigma_{2}^{2}/{\left(x_{1}-x_{2}\right)}^{2}$, $z_{1}=v_{1}s_{1}^{2}/{\left(x_{1}-x_{2}\right)}^{2}$ and $z_{2}=v_{2}s_{2}^{2}/{\left(x_{1}-x_{2}\right)}^{2}$, under lexicographic order, $\sigma_{1}^{2}>\sigma_{2}^{2}$, the Groebner basis for equations (13) written in the form

(14) $$u^{2}+\left(z_{1}-n_{1}u\right){\left(u+\upsilon \right)}^{2}=0,$$

(15) $$\upsilon^{2}+\left(z_{2}-n_{2}\upsilon \right){\left(u+\upsilon \right)}^{2}=0,$$

consists of two polynomials,

(16) $$\begin{array}{c} G_{1}\left(u,\upsilon \right)=c_{0}u^{2}+c_{1}\upsilon^{5}+c_{2}\upsilon^{4}+c_{3}\upsilon^{3}+c_{4}\upsilon^{2},\\ c_{0}=n_{1}^{2}z_{2}^{3}\left[f\left(1+z_{1}\right)+n_{1}z_{2}\right],\\ c_{1}=-n^{2}n_{2}^{3}f\;z_{1}+n_{1}n_{2}\left(n_{1}+3n_{2}\right)n^{3}z_{2}-n^{2}n_{2}^{3}f,\\ c_{2}=-2n_{2}^{3}n\;f\;z_{1}^{2}+2n_{2}n\left(n_{1}^{3}+4n_{1}^{2}n_{2}+4n_{1}n_{2}^{2}+2n_{2}^{3}\right)z_{1}z_{2}\\ -n_{1}n\left(n_{1}^{2}n_{2}+4n_{1}n_{2}+6n_{2}^{2}\right)z_{2}^{2}+f\;n_{1}^{2}n_{2}^{2}z_{1}\\ -n\left(5n_{1}^{4}+5n_{1}^{3}n_{2}+8n_{1}^{2}n_{2}^{2}+4n_{1}^{2}n_{2}^{3}-4n_{2}^{4}\right)z_{2}\\ +f\;n_{2}^{2}\left(n_{1}^{2}+2n_{1}n_{2}+2n_{2}^{2}\right),\\ c_{3}=-f\;n_{2}^{3}z_{1}^{3}+n_{2}\left(n_{1}^{3}+4n_{1}^{2}n_{2}+5n_{1}n_{2}^{2}+4n_{2}^{3}\right)z_{1}^{2}z_{2}\\ -\left(2n_{1}^{4}+8n_{1}^{3}n_{2}+12n_{1}^{2}n_{2}^{2}+7n_{1}n_{2}^{3}+2n_{2}^{4}\right)z_{1}z_{2}^{2}\\ +3n_{1}n_{2}n^{2}z_{2}^{3}-3\;f\;n_{2}^{3}z_{1}^{2}+\left(3n_{1}^{3}n_{2}+10n_{1}^{2}n_{2}^{2}+6n_{1}n_{2}^{3}\right)z_{1}z_{2}^{2}\\ +n_{2}n\left(5n_{1}^{2}+7n_{1}n_{2}-2n_{2}^{2}\right)z_{2}^{2}\\ +3\;f\;n_{2}^{3}z_{1}+n_{2}\left(2n_{1}^{3}+6n_{1}^{2}n_{2}+n_{1}n_{2}^{2}-4n_{2}^{3}\right)z_{2}-f\;n_{2}^{3},\\ c_{4}=-n_{1}^{2}z_{1}z_{2}^{2}\left[f\;\left(1+z_{1}\right)+n_{1}z_{2}\right], \end{array}$$

and

(17) $$\begin{array}{c} G_{2}\left(u,\upsilon \right)=d_{0}u\upsilon +d_{1}\upsilon^{5}+d_{2}\upsilon^{4}+d_{3}\upsilon^{3}+d_{4}\upsilon^{2},\\ d_{0}=2n_{1}z_{2}^{2}\left[f\left(1+z_{1}\right)+n_{1}z_{2}\right],\\ d_{1}=-n_{2}f^{2}n^{2},\\ d_{2}=-2nn_{2}f^{2}z_{1}+n\;f\left(n_{1}^{2}+3n_{1}n_{2}+4n_{2}^{2}\right)z_{2}\\ +f^{2}\left(n_{1}^{2}+2n_{1}n_{2}+2n_{2}^{2}\right),\\ d_{3}=-n_{2}f^{2}z_{1}^{2}+2f\left(n_{1}^{2}+2n_{1}n_{2}+2n_{2}^{2}\right)z_{1}z_{1}\\ -n_{2}\left(3n_{1}^{2}+6n_{1}n_{2}+4n_{2}^{2}\right)z_{2}^{2}-2n_{2}f^{2}z_{1}-4nn_{2}fz_{2}-f\;n_{2}^{3},\\ d_{4}=n_{1}z_{2}\left(1+z_{1}+z_{2}\right)\left[f\left(1+z_{1}\right)+n_{1}z_{2}\right]. \end{array}$$

This fact can be derived from the definition of the Groebner basis and confirmed by existing computational algebra software.

It follows that for stationary points *u*, *υ*, *G*_1_(*u*, *υ*) = *G*_2_(*u*, *υ*) = 0. All these points can be found by expressing *u* through *υ* via (17),

(18) $$u=-\upsilon \left(d_{1}\upsilon^{3}+d_{2}\upsilon^{2}+d_{3}\upsilon +d_{4}\right)/d_{0},$$

substituting this expression in (16), and solving then the resulting sextic equation for *υ*,

(19) $$\begin{array}{l} c_{0}{\left(d_{1}\upsilon^{3}+d_{2}\upsilon^{2}+d_{3}\upsilon +d_{4}\right)}^{2}\\ \;+d_{0}^{2}\left(c_{1}\upsilon^{3}+c_{2}\upsilon^{2}+c_{3}\upsilon +c_{4}\right)=0. \end{array}$$

Thus, there are 3 × 6 = 18 complex root pairs out of which positive roots *u*, *υ* are to be chosen. Although in practice most all these roots are complex or negative and are not meaningful, sometimes the number of positive roots is fairly large. For example, *x*_1_ = −0.391, *x*_2_ = 0.860, $s_{1}^{2}=0.075$, $s_{2}^{2}=0.102$, *n*_1_ = *n*_2_ = 3, there are three positive solutions, (*u* = 0.263, *υ* = 0.053), (*u* = 0.138, *υ* = 0.106), (*u* = 0.035, *υ* = 0.312). In this particular case one has to compare the likelihood function evaluated at these solutions with the likelihood evaluated when ${\hat{\sigma }}^{2}>0$, in which case the stationary points are (*u* = *υ* = 0.065, *z* = 0.230), (*u* = 0.246, *υ* = 0.055, *z* = 0.003), (*u* = 0.042, *υ* = 0.234, *z* = 0.019), (*u* = 0.048, *υ* = 0.065, *z* = 0.193). (The likelihood is maximized at the last solution.)

If ${\hat{\sigma }}^{2}=0$, the estimators ${\hat{\sigma }}_{1}^{2}$, ${\hat{\sigma }}_{2}^{2}$ satisfy the equations,

(20) $$\frac{{\left(x_{1}-x_{2}\right)}^{2}}{{\left(\sigma_{1}^{2}+\sigma_{2}^{2}\right)}^{2}}=\frac{n_{1}}{\sigma_{1}^{2}}-\frac{v_{1}s_{1}^{2}}{\sigma_{1}^{4}}=\frac{n_{2}}{\sigma_{2}^{2}}-\frac{v_{2}s_{2}^{2}}{\sigma_{2}^{4}},$$

which imply the inequalities $v_{i}s_{i}^{2}/n_{i}<{\hat{\sigma }}_{i}^{2}<\left[{\left(x_{1}-x_{2}\right)}^{2}+v_{i}s_{i}^{2}\right]/n_{i}$. Evaluation of second derivatives shows that $2n{\hat{\sigma }}_{1}^{2}{\hat{\sigma }}_{2}^{2}{\left(x_{1}-x_{2}\right)}^{2}\leq n_{1}n_{2}\left({\hat{\sigma }}_{1}^{2}+{\hat{\sigma }}_{2}^{2}\right)$.

These solutions are to be compared with the solutions when ${\hat{\sigma }}^{2}>0$, in which case with *y* = *σ*^2^/(*x*_1_−*x*_2_)^2^ > 0,

(21) $${\left(2y+u+\upsilon \right)}^{3}-2\left(y+u\right)\left(y+\upsilon \right)=0,$$

(22) $$u^{2}\left(u-\upsilon \right)+2\left(z_{1}-v_{1}u\right)\left(y+u\right)\left(y+\upsilon \right)=0,$$

(23) $$\upsilon^{2}\left(\upsilon -u\right)+2\left(z_{2}-v_{2}\upsilon \right)\left(y+u\right)\left(y+\upsilon \right)=0.$$

Notice that (21)–(23) imply that if ${\hat{\sigma }}^{2}>0$, then the inequalities $s_{1}^{2}\geq {\hat{\sigma }}_{1}^{2}$, $s_{2}^{2}\leq {\hat{\sigma }}_{2}^{2}$, and ${\hat{\sigma }}_{1}^{2}\leq {\hat{\sigma }}_{2}^{2}$ are all equivalent. The fact excludes the possibility that ${\hat{\sigma }}_{i}^{2}=s_{i}^{2}$, unless $s_{1}^{2}=s_{2}^{2}$, in which case ${\hat{\sigma }}_{1}^{2}={\hat{\sigma }}_{2}^{2}=s_{1}^{2}$, ${\hat{\sigma }}^{2}={\left[{\left(x_{1}-x_{2}\right)}^{2}/4-s_{1}^{2}\right]}_{+}$, *x*_+_ = max(0, *x*). In this and only in this situation (whose probability is zero), ${\hat{\sigma }}_{i}^{2}=s_{i}^{2}$. The equation (21) implies that 2 *y* + *u* + *υ* ≤ ½, so that

(24) $${\hat{\sigma }}^{2}\leq \frac{{\left(x_{1}-x_{2}\right)}^{2}}{4}.$$

Unfortunately, the Groebner basis for equations (21)–(23) has a much more complicated structure: the form of the monomials entering into the basis polynomials depends on z_1_, z_2_, n_1_ and n_2_. To find solutions to (21)–(23) we start with conditions on *u* and *υ* for which *y* > 0 (21)–(23).

For fixed *u* and *υ* the behavior of the derivative of (9) with respect to *σ*^2^ is determined by that of the cubic polynomial (12) which now takes the form,

(25) $$F\left(y\right)={\left(2y+u+\upsilon \right)}^{3}-2\left(y+u\right)\left(y+\upsilon \right).$$

This derivative is positive if and only if *F*(*y*) > 0.

As is easy to check, the derivative of *F* has two roots: −(*u* + *υ*)/2 and y* = 1/6 − (*u* + *υ*)/2. The polynomial *F* has no positive roots if and only if either y* ≤ 0, *F*(0) ≥ 0 or if y* > 0, *F*(y*) ≥ 0. We rewrite these conditions as follows: (21) has a positive root if and only if either

(26) $$u+\upsilon \geq \frac{1}{3},{\left(u+\upsilon \right)}^{3}<2u\upsilon ,$$

or

(27) $$u+\upsilon <\frac{1}{3},|u-\upsilon |<\frac{1}{3\sqrt{3}}.$$

If (26) holds, there is a unique positive stationary point. When condition (27) is met, only the largest root of *F*(*y*) = 0 (i.e., the one exceeding y*) can be the ML estimator ${\hat{\sigma }}^{2}$. Analysis of second derivatives shows that (*y*, *u*, *υ*), *y* > 0, can provide the minimum only if $2y+u+\upsilon >\sqrt{3}|u-\upsilon |$ and (*y* + *u*) (*y + υ*) min (*v*_1_(*y* + *u*)/*u*, (*v*_2_(*y* + *υ*)/*υ*, ≥ *y* |*u* − *υ*|.

**Fig. 1:**
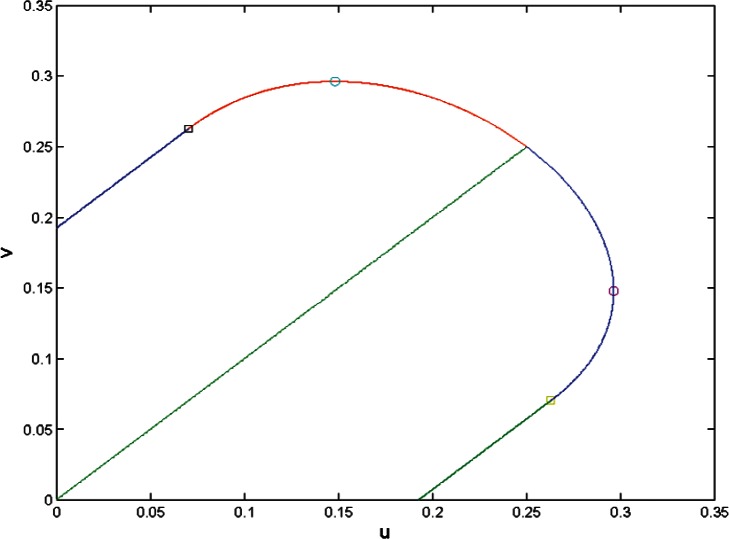
The region where (21) has a positive root. The squares mark the points where the boundary changes from linear to cubic. Two points of this region at which *u* or *υ* take the largest possible value (8/27) are marked by *o*.

Figure 1 shows the region where (21) has a positive root in the (*u*, *υ*) plane. Its boundary is formed by two straight segments where $3\sqrt{3}|u-\upsilon |=1$, 3(*u* + *υ*) ≥ 1, and by a cubic curve when *u* + *υ* ≥ 1/3. The largest possible value of *u* or *υ* such that ${\hat{\sigma }}^{2}>0$ is 8/27.

To study further the form of solutions to (21)–(23), we use the fact that

(28) $$\begin{array}{l} \arg\;\min\;L\\ =\arg\;\underset{z_{1}/u+z_{2}/v+1/(2y+u+v)=n}{\min}\\ \left[v_{1}\log u+v_{2}\log\upsilon +\log\left(y+u\right)+\log\left(y+\upsilon \right)\right], \end{array}$$

which can be shown by the direct calculation or by the argument from Sec. 2.2. Thus assuming the condition *z*_1_ / *u* + *z*_2_ / *υ* + 1/(2*y* + *u* + *υ*) = *n*, one can parametrize the solutions of Eqs. (21)–(23) as follows: for *s* in the unit interval, *u* = (*z*_1_*s* + *z*_2_ (1 − *s*))/[*s*(*n* − 1/*K*)], *υ* = (*z*_1_*s* + *z*_2_(1 − *s*))/[(1 − *s*)(*n* − 1/*K*)], *u* + *y*= 2 *w*(1 − *w*)^2^, *υ* + *y* = 2*w*^2^(1 − *w*) with *K* = 2 *y* + *u* +*υ* = 2 *w*(1 − *w*) > 1/*n*, where *w*, 0 < *w* < 1, solves the cubic equation *u* − *υ* = 2(1 − 2*w*)*w*(1 − *w*) or

(29) $$\begin{array}{l} {\left(1-2w\right)}^{3}-\frac{\left(n-2\right)\left(1-2w\right)}{n}+\\ \;\frac{2\left(1-2s\right)\left(z_{1}s+z_{2}\left(1-s\right)\right)}{ns\left(1-s\right)}=0. \end{array}$$

The conditions (26) and (27) reduce to the inequalities,

(30) $$2s\left(1-s\right)\left(n-\frac{1}{K}\right)\geq \frac{z_{1}s+z_{2}\left(1-s\right)}{ns\left(1-s\right)},$$

and

(31) $$\left|1-2w\right|w\left(1-w\right)<\frac{1}{6\sqrt{3}}.$$

The discriminant of the cubic equation (29) reveals that when *s* ≠ 1/2, $|1-2\;s|\left[z_{1}s+z_{2}\left(1-s\right)\right]<{\left(n-2\right)}^{3/2}s\left(1-s\right)/\left(3\sqrt{3n}\right)$, and (29) has three real roots, two of them of the same sign as 1 − 2 *s*. *If s* = 1/2, then we have *w* = 1/2. Indeed the condition *y* ≥ 0 leads to the following restriction on the range of *s*: | 1 − 2 *w* | ≤ | 1 − 2 *s*| with a strict inequality when *y* > 0.

The end points of this domain can be found as solutions to the equation,

(32) $$\begin{array}{l} s\left(1-s\right){\left(1-2s\right)}^{2}-\\ \;\frac{\left(n-2\right)s\left(1-s\right)}{n}+\frac{2\left(z_{1}s+z_{2}\left(1-s\right)\right)}{n}=0, \end{array}$$

which is a quartic equation in *ξ* = 1−2*s*,

(33) $$\xi^{4}-\frac{2\left(n-1\right)}{n}\xi^{2}-\frac{8\Delta }{n}\xi -\frac{8\bar{z}-n+2}{n}=0.$$

Here $\bar{z}=\left(z_{1}+z_{2}\right)/2,\Delta =\left(z_{2}-z_{1}\right)/2,|\Delta |<\bar{z}$.

If the equation (33) has two distinct roots $\underline{\xi },\bar{\xi }$ in the interval (−1, 1), then the range of *s*-values is the interval $\left[\underline{s},\bar{s}\right]$, $0<\underline{s}=\left(1-\bar{\xi }\right)/2<\bar{s}=\left(1-\underline{\xi }\right)/2<1$, and for *s* ∈ $\left(\underline{s},\bar{s}\right)$ the polynomial in (32) is negative. Therefore, if there are two roots of (29) in the interval (0, 1) which have the same sign as 1 − 2*s*, the one with the smallest absolute value of |1 − 2*w*|, i.e., the one with $|1-2w|<\sqrt{\left(n-2\right)/\left(3n\right)}$, is to be chosen. Thus, the solutions of equations (21)–(23) are parameterized by *s*∈ $\left[\underline{s},\bar{s}\right]$.

To determine conditions for the equation (33) to have two roots $\underline{\xi },\bar{\xi }$, in the interval (−1, 1), observe first that it cannot have more than two roots there. Indeed the polynomial in (33) assumes negative values at the end points, and it has exactly two roots if and only if there is a point in this region at which that polynomial is positive.

If the derivative of this polynomial, 4*ξ*^3^ − 4 (*n* − 1) *ξ*/*n* − 8Δ/*n*, does not vanish in (−1, 1), then (33) cannot have two roots there. This happens when this derivative has just one real root, or equivalently the discriminant of the cubic polynomial is negative which means that | Δ | > [(*n* − 1)/(3*n*)]^3/2^ and which implies that | Δ | > 1/2. Then the derivative takes negative values at the end points of the interval (−1, 1).

If *ξ*_0_ is the point of maximum of the polynomial (33) in (−1, 1), then $4\xi_{0}^{3}-4\left(n-1\right)\xi_{0}/n=8\Delta /n$, and the roots $\underline{\xi },\bar{\xi }$ exist if and only if

(34) $$\begin{array}{r} \xi_{0}^{4}-\frac{2\left(n-1\right)}{n}\xi_{0}^{2}-\frac{8\Delta }{n}\xi_{0}-\frac{8\bar{z}-n+2}{n}=\\ 3\xi_{0}^{4}+\frac{2\left(n-1\right)}{n}\xi_{0}^{2}-\frac{8\bar{z}-n+2}{n}>0. \end{array}$$

According to inequality (34),

(35) $$\left|\xi_{0}^{2}-\frac{n-1}{3n}\right|\leq \sqrt{\frac{4n^{2}-8n+1-24n\bar{z}}{9n^{2}}},$$

and (35) shows that $\bar{z}\leq \left(4n^{2}-8n+1\right)/\left(24\;n\right)\leq {\left(n-1\right)}^{3/2}/\left(3\sqrt{3n}\right)$. One must have $|\xi_{0}|\leq \sqrt{\left(n-1\right)/\left(3\;n\right)}$, as the derivative decreases only in this sub-interval of (−1, 1). Thus

(36) $$\xi_{0}^{2}\geq \frac{n-1}{3n}\left[1-\sqrt{\frac{4n^{2}-8n+1-24n\bar{z}}{{\left(n-1\right)}^{2}}}\right].$$

If $8\bar{z}<n-2\left(\text{i.e.},4n^{2}-8n+1-24n\bar{z}>{\left(n-1\right)}^{2}\right)$, Eq. (33) must have two roots $\underline{\xi },\bar{\xi }$ as it takes a positive value when *ξ* = 0. Since sign(*ξ*_0_) = −sign (Δ), assuming that $8\bar{z}\geq n-2$, we get

(37) $$\begin{array}{l} \frac{2\left|\Delta \right|}{n}=\left(\frac{n-1}{n}-\xi_{0}^{2}\right)\left|\xi_{0}\right|\\ \;\geq {\left(\frac{n-1}{3n}\right)}^{3/2}\left[2+\sqrt{\frac{4n^{2}-8n+1-24\;n\bar{z}}{{\left(n-1\right)}^{2}}}\right]\\ \;{\left[1-\sqrt{\frac{4n^{2}-8n+1-24n\bar{z}}{{\left(n-1\right)}^{2}}}\right]}^{1/2}, \end{array}$$

which means that

(38) $$\begin{array}{l} {\left(4n^{2}-8n+1-24n\bar{z}\right)}^{3/2}\\ \;+3\left(n-1\right)\left(4n^{2}-8n+1-24n\bar{z}\right)\\ \;\geq 4\left[{\left(n-1\right)}^{3}-27n\Delta^{2}\right]\geq 0. \end{array}$$

The condition (38) can also be shown to be sufficient for the existence of $\underline{\xi },\bar{\xi }$.

**Fig. 2:**
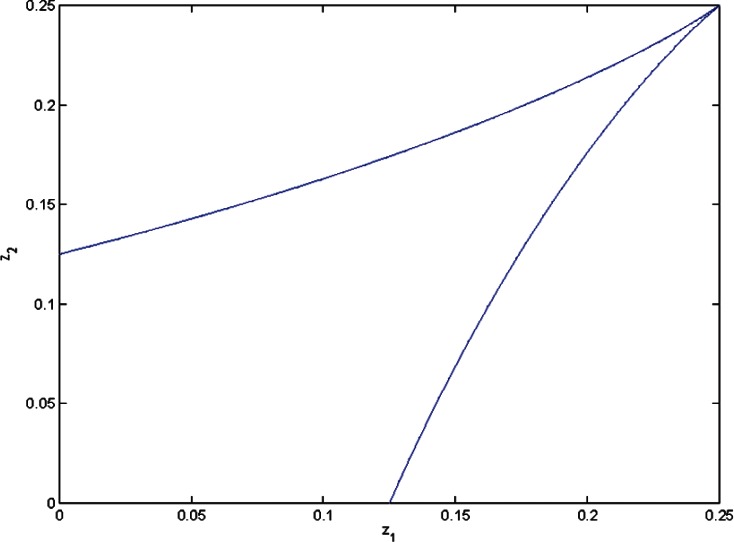
The region where ${\hat{\sigma }}^{2}>0$ when *n*_1_= 2, *n*_2_= 2.

**Fig. 3:**
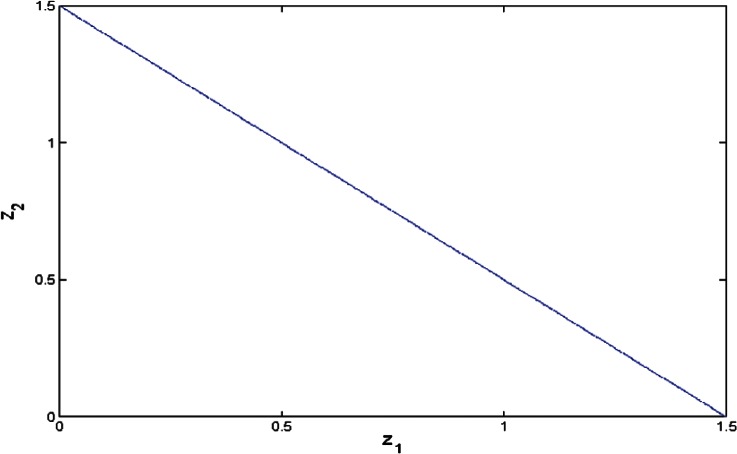
The region where ${\hat{\sigma }}^{2}>0$ when *n*_1_= 4, *n*_2_= 4.

**Fig. 4:**
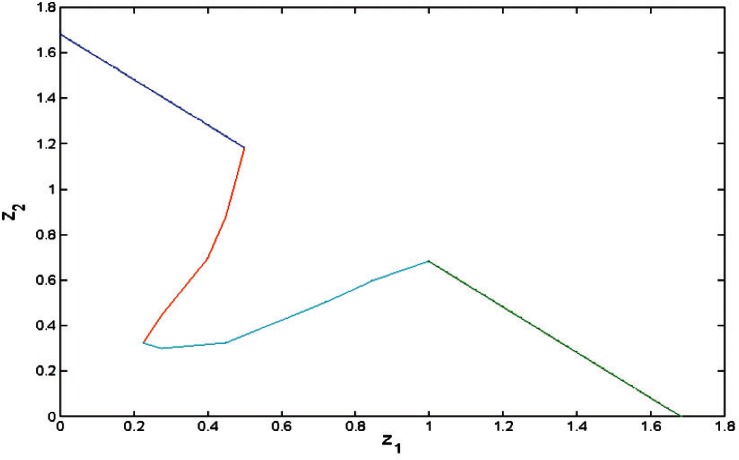
The region where ${\hat{\sigma }}^{2}>0$ when *n*_1_= 8, *n*_2_= 3.

This parametrization and the Groebner basis for (13) allow for given *z*_1_, *z*_2_ to compare the minimal values of (9) when *y* > 0 with that when *y* = 0. Figures 2–4 show bounded regions where ${\hat{\sigma }}^{2}>0$ in the space (*z*_1_, *z*_2_) when *n*_1_ = *n*_2_ = 2, *n*_1_ = *n*_2_ = 4, or *n*_1_ = 8, *n*_2_ = 3. When *n*_1_ = *n*_2_ ≥ 3, this region is a triangle, *z*_1_ + *z*_2_ < *c*; for *n*_1_ ≠ *n*_2_ it is more complicated.

We summarize the obtained results.

**Theorem 2.1.** *When p* = 2, *the Groebner basis for (13) is given by (16), (17). The solutions of (21)–(23) satisfy conditions (26) and (27). These solutions can be parametrized via (29) by s* ∈ $\left(\underline{s},\bar{s}\right)$ *where the end points can be found from (32) given that (38) holds.*

We conclude by noticing the relationship of the problem discussed here to the likelihood solutions to the classical Behrens-Fisher problem [10] which assumes that *σ*^2^ = 0.

### 2.2 Solving Likelihood Equations Numerically

In view of the difficulty in evaluating the Groebner basis for *p* > 2, an iterative method to solve the optimization problem in (2) is of interest. Notice that the sum of the first two terms in (2) is a homogeneous function of *σ*^2^, $\sigma_{1}^{2},\ldots ,\sigma_{p}^{2}$, of degree −1. Therefore, with *n* = ∑*_i_ n_i_*

(39) $$\begin{array}{l} \underset{\sigma^{2},\sigma_{1}^{2},\ldots ,\sigma_{p}^{2}}{\min}L=\underset{\lambda \sigma^{2},\lambda \sigma_{1}^{2},\ldots ,\lambda \sigma_{p}^{2}}{\min}L\\ \;=\underset{\lambda ,\sigma^{2},\sigma_{1}^{2},\ldots ,\sigma_{p}^{2}}{\min}[\sum_{i}\frac{{\left(x_{i}-\bar{\mu }\right)}^{2}}{\lambda \left(\sigma^{2}+\sigma_{i}^{2}\right)}+\sum_{i}\frac{v_{i}s_{i}^{2}}{\lambda \sigma_{i}^{2}}\\ \;+\sum_{i}\log\left(\lambda \left(\sigma^{2}+\sigma_{i}^{2}\right)\right)+\sum_{i}v_{i}\log\left(\lambda \sigma_{i}^{2}\right)]\\ \;=\underset{\sigma^{2},\sigma_{1}^{2},\ldots ,\sigma_{p}^{2}}{\min}[\underset{\lambda }{\min}[\frac{1}{\lambda }(\sum_{i}\frac{{\left(x_{i}-\bar{\mu }\right)}^{2}}{\sigma^{2}+\sigma_{i}^{2}}\\ \;+\sum_{i}\frac{v_{i}s_{i}^{2}}{\sigma_{i}^{2}})+n\;\log\lambda ]+\sum_{i}\log\left(\sigma^{2}+\sigma_{i}^{2}\right)\\ \;+\sum_{i}v_{i}\log\sigma_{i}^{2}]\\ \;=\underset{y_{0},y_{1},\ldots ,y_{p}}{\min}[n\;\log\left(\sum_{i}\frac{{\left(x_{i}-\bar{\mu }\right)}^{2}}{y_{0}+y_{i}}+\sum_{i}\frac{v_{i}s_{i}^{2}}{y_{i}}\right)\\ \;+\sum_{i}\log\left(y_{0}+y_{i}\right)+\sum_{i}v_{i}\log y_{i}]\\ \;+n-n\log n. \end{array}$$

The minimizing value of *λ* is $\lambda_{0}=\left(\sum_{i}{\left(x_{i}-\bar{\mu }\right)}^{2}/\left(y_{0}+y_{i}\right)+\sum_{i}v_{i}s_{i}^{2}/y_{i}\right)/n$. Thus the objective function in (39) becomes homogeneous of degree 0 in, *y*_0_, *y*_1_, …, *y_p_* which reduces the problem to minimization of such a function. If *y*_0_, *y*_1_, …, *y_p_* is a solution to the problem (39), then ${\hat{\sigma }}^{2}=\lambda_{0}y_{0}$, ${\hat{\sigma }}_{1}^{2}=\lambda_{0}y_{1},\ldots ,{\hat{\sigma }}_{p}^{2}=\lambda_{0}y_{p}$ is a ML solution.

To minimize the objective function in (39)

(40) $$\begin{array}{l} F\left(y_{0},y_{1,\ldots ,}y_{p}\right)\\ \;=n\log\left(\sum_{i}\frac{{\left(x_{i}-\bar{\mu }\right)}^{2}}{y_{0}+y_{i}}+\sum_{i}\frac{v_{i}s_{i}^{2}}{y_{i}}\right)\\ \;+\sum_{i}\log\left(y_{0}+y_{i}\right)+\sum_{i}v_{i}\log y_{i}, \end{array}$$

one can impose a restriction on *y*_0_, *y*_1_, …, *y_p_* such as ∑ *y_i_* = 1 or *λ*_0_ = 1. Note that *F* tends to +∞ when *y_i_* → 0 or *y_i_* → +∞ for any fixed *y*_0_ > 0. Since $\sum_{i}\left(x_{i}-\bar{\mu }\right)/\left(y_{0}+y_{i}\right)=0$, one sees that

(41) $$\frac{\partial }{\partial y_{i}}F=-n\frac{\frac{{\left(x-\bar{\mu }\right)}^{2}}{{\left(y_{0}+y_{i}\right)}^{2}}+\frac{v_{i}s_{i}^{2}}{y_{i}^{2}}}{\sum_{j}\left[\frac{{\left(x_{j}-\bar{\mu }\right)}^{2}}{y_{0}+y_{j}}+\frac{v_{j}s_{j}^{2}}{y_{j}}\right]}+\frac{1}{y_{0}+y_{i}}+\frac{v_{i}}{y_{i}},$$

which must be positive for large *y_i_* and negative for sufficiently small *y_i_*.

If *y*_0_ > 0, by equating this derivative to zero, we obtain

(42) $$\frac{s_{i}^{2}}{y_{i}^{2}}-\frac{\lambda_{0}}{y_{i}}+\frac{\lambda_{i}-n_{i}\lambda_{0}}{v_{i}y_{0}}=0,$$

where $\lambda_{i}={\left(x_{i}-\bar{\mu }\right)}^{2}/\left(y_{0}+y_{i}\right)+v_{i}s_{i}^{2}/y_{i}$ so that Σ*λ_i_* = *nλ*_0_. The equation (42) must have a positive root, which means that for *λ_i_* evaluated at a stationary point,

(43) $$\frac{v_{i}y_{0}}{4s_{i}^{2}}\geq \frac{\lambda_{i}-n_{i}\lambda_{0}}{\lambda_{0}^{2}},$$

and

(44) $$\frac{s_{i}}{y_{i}}=\frac{\lambda_{0}}{2s_{i}}+\sqrt{\frac{\lambda_{0}^{2}}{4s_{i}^{2}}+\frac{n_{i}\lambda_{0}-\lambda_{i}}{v_{i}y_{0}}}<\frac{\lambda_{0}}{2s_{i}}+\sqrt{\frac{\lambda_{0}^{2}}{4s_{i}^{2}}+\frac{n_{i}\lambda_{0}}{v_{i}y_{0}}}.$$

It follows that for any stationary point, $2s_{i}^{2}>\lambda_{0}y_{i}$, i.e.,

(45) $$\frac{2s_{i}^{2}}{1+\sqrt{1+\frac{4n_{i}s_{i}^{2}}{v_{i}{\hat{\sigma }}^{2}}}}<{\hat{\sigma }}_{i}^{2}<2s_{i}^{2}.$$

For a fixed value of *λ*_0_, say, *λ*_0_ = 1, the Eq. (42) leads to an iteration scheme, with specified initial values of *y*_0_^(0)^ and ${\bar{\mu }}_{0}$. We take these to be the estimates arrived at by the method of moments as described later in Sec. 5, but once they are given, one can solve the cubic equation for *υ_i_* = *y*_0_ / *y_i_*,

(46) $$\begin{array}{l} \upsilon_{i}{\left(1+\upsilon_{i}\right)}^{2}v_{i}s_{i}^{2}-{\left(1+\upsilon_{i}\right)}^{2}v_{i}y_{0}\\ \;-\left(1+\upsilon_{i}\right)y_{0}+v_{i}{\left(x_{i}-\bar{\mu }\right)}^{2}=0. \end{array}$$

It is easy to see that each of these equations has either one or three positive roots. If there is just one root, then it uniquely defines *υ_i_*. In case of three positive roots, the root which minimizes (40) is chosen. Under this agreement, at stage *k* in (46) *υ_i_* = *υ_i_*^(^*^k^*^)^, *y*_0_ = *y*_0_^(^*^k^*^)^, $\bar{\mu }={\bar{\mu }}_{k}$, and after solving (46) we put

(47) $${\bar{\mu }}_{k+1}=\frac{\sum_{j}\frac{\upsilon_{j}^{\left(k+1\right)}x_{j}}{1+\upsilon_{j}^{\left(k+1\right)}}}{\sum_{j}\frac{\upsilon_{j}^{\left(k+1\right)}}{1+\upsilon_{j}^{\left(k+1\right)}}},$$

and

(48) $$\begin{array}{l} y_{0}^{\left(k+1\right)}=\frac{1}{n}\left[\sum_{i}\frac{\upsilon_{i}^{\left(k+1\right)}{\left(x_{i}-{\bar{\mu }}_{k+1}\right)}^{2}}{1+\upsilon_{i}^{\left(k+1\right)}}+\sum_{i}v_{i}s_{i}^{2}\upsilon_{i}^{\left(k+1\right)}\right],\\ k=\;0,\;1,\;\ldots . \end{array}$$

As in [11], (46) defines a sequence converging to a stationary point, and at each step the value of (40) is decreased.

**Theorem 2.2** *Successive-substitution iteration defined by equations (46), (47) and (48) converges to a stationary point of (40) so that at each step this function decreases. At any stationary point inequalities (43) and (44) hold and (45) is valid.*

Notice that Vangel and Rukhin tacitly assume in [11] that *y*_0_ > 0. The case of the global minimum attained at the boundary, i.e., when *y*_0_ = 0, can be handled as follows. Equating (41) to zero gives

(49) $$y_{i}\propto \frac{{\left(x_{i}-\bar{\mu }\right)}^{2}+v_{i}s_{i}^{2}}{n_{i}},$$

and a simpler version of an iterative scheme

(50) $$\begin{array}{c} y_{i}^{\left(k+1\right)}=\frac{\frac{{\left(x_{i}-{\bar{\mu }}_{k}\right)}^{2}+v_{i}s_{i}^{2}}{n_{i}}}{\sum_{j}\frac{{\left(x_{j}-{\bar{\mu }}_{k}\right)}^{2}+v_{j}s_{j}^{2}}{y_{j}^{\left(k\right)}}},{\bar{\mu }}_{k+1}\\ =\sum_{j}\frac{x_{j}}{y_{j}^{\left(k+1\right)}}{\left[\sum_{j}\frac{1}{y_{j}^{\left(k+1\right)}}\right]}^{-1}, \end{array}$$

*k* = 0, 1, …, $y_{j}^{\left(0\right)}=s_{j}^{2}$, *y*_0_ = 0, converges fast. However the solutions obtained via (46), (47), (48) and (50) are to be compared by evaluating *L*. To assure that a global minimum has been found, several starting values should be tried.

The maximum likelihood estimator and the restricted maximum likelihood estimator discussed in the next section can be computed via their *R*-language implementation through the *lme* function from *nlme* library [12]. However this routine has a potential (false or singular) convergence problem occurring in some practical situations.

## 3. Restricted Maximum Likelihood

The possible drawback of the maximum likelihood method is that it may lead to biased estimators. Indeed the maximum likelihood estimators of *σ* ’s do not take into account the loss in degrees of freedom that results from estimating *μ*. This undesirable feature is eliminated in the restricted maximum likelihood (REML) method which maximizes the likelihood corresponding to the distribution associated with linear combinations of observations whose expectation is zero. This method discussed in detail by Harville [13] has gained considerable popularity and is employed now as a tool of choice by many statistical software packages.

The negative restricted likelihood function has the form

(51) $$\begin{array}{l} RL=L+\log\left(\sum_{i}{\left(\sigma^{2}+\sigma_{i}^{2}\right)}^{-1}\right)=\\ \;\sum_{1\leq i<j\leq p}\frac{{\left(x_{i}-x_{j}\right)}^{2}}{\left(\sigma^{2}+\sigma_{i}^{2}\right)\left(\sigma^{2}+\sigma_{j}^{2}\right)}{\left[\sum_{i}{\left(\sigma^{2}+\sigma_{i}^{2}\right)}^{-1}\right]}^{-1}\\ +\log\left(\sum_{i}{\left(\sigma^{2}+\sigma_{i}^{2}\right)}^{-1}\right)+\sum_{i}\log\left(\sigma_{i}^{2}+\sigma^{2}\right)\\ \;+\sum_{i}\frac{v_{i}s_{i}^{2}}{\sigma_{i}^{2}}+\sum_{i}v_{i}\log\sigma_{i}^{2}. \end{array}$$

By using notation (5) and (6), we can rewrite

(52) $$RL=\frac{Q}{P^{\prime }}+\log P^{\prime }+\sum_{i}\frac{v_{i}s_{i}^{2}}{\sigma_{i}^{2}}+\sum_{i}v_{i}\log\sigma_{i}^{2}.$$

To estimate *σ*^2^ and $\sigma_{i}^{2}$, *i* = 1, …, *p* one has to find the minimal value of *Q*/*P*′ + log *P*′. The derivative of this function in *σ*^2^ is proportional to the polynomial *H* of degree 2*p*−3 in this variable,

(53) $$H=P^{\prime }P^{\prime \prime }+Q^{\prime }P^{\prime }-QP^{\prime \prime },$$

as opposed to 3*p*−3 which is the degree of the corresponding polynomial *F* in (12) under the ML scenario.

For fixed $\sigma_{i}^{2}$, *i* = 1, …, *p*, all *p* roots of the polynomial *P* = *P* (*σ*^2^) are real numbers, so that

(54) $$PP^{\prime \prime }\leq \frac{p-1}{p}{\left[P^{\prime }\right]}^{2}\leq {\left[P^{\prime }\right]}^{2}$$

[14, 3.3.29].

An application of (54) shows that

(55) $$F\geq P\left(P^{\prime }P^{\prime \prime }+Q^{\prime }P^{\prime }-QP^{\prime \prime }\right)=PH.$$

Since *P* (*σ*^2^) > 0, if *F* has a positive root *σ*^2^, then *H* also must have a positive root *τ*^2^. Moreover, *τ*^2^ ≤ *σ*^2^, so that the ML estimator of *σ*^2^ is always smaller than the REML estimator of the same parameter for the same data.

The polynomial equations for the restricted likelihood method have the form,

(56) $$\sigma_{i}^{4}\left({Q^{\prime }}_{i}P^{\prime }-Q{P^{\prime }}_{i}+{P^{\prime }}_{i}P^{\prime }\right)+v_{i}\left(\sigma_{i}^{2}-s_{i}^{2}\right){\left(P^{\prime }\right)}^{2}=0,$$

with each of these polynomials being of degree 3 in $\sigma_{i}^{2}$, *i* = 1, …, *p*. When *σ*^2^ > 0, Eqs. (56) have to be augmented by the equation *H* = 0.

### 3.1 Solving REML Equations

To solve the optimization problem in (51) in practice one can use a method similar to that in Sec. 2.2. Indeed,

(57) $$\begin{array}{l} \underset{\sigma^{2},\sigma_{1}^{2},\ldots ,\sigma_{p}^{2}}{\min}RL=\underset{\lambda ,\sigma^{2},\sigma_{1}^{2},\ldots ,\sigma_{p}^{2}}{\min}[\sum_{i}\frac{{\left(x_{i}-\bar{\mu }\right)}^{2}}{\lambda \left(\sigma^{2}+\sigma_{i}^{2}\right)}+\sum_{i}\frac{v_{i}s_{i}^{2}}{\lambda \sigma_{i}^{2}}\\ +\log\left(\sum_{i}\frac{1}{\lambda \left(\sigma^{2}+\sigma_{i}^{2}\right)}\right)+\sum_{i}\log\left(\lambda \left(\sigma^{2}+\sigma_{i}^{2}\right)\right)\\ +\sum_{i}v_{i}\log\left(\lambda \sigma_{i}^{2}\right)]\\ =\underset{\sigma^{2},\sigma_{1}^{2},\ldots \sigma_{p}^{2}}{\min}[\underset{\lambda }{\min}[\frac{1}{\lambda }\left(\sum_{i}\frac{{\left(x_{i}-\bar{\mu }\right)}^{2}}{\sigma^{2}+\sigma_{i}^{2}}+\sum_{i}\frac{v_{i}s_{i}^{2}}{\sigma_{i}^{2}}\right)\\ +\left(n-1\right)\log\lambda ]+\log\left(\sum_{i}\frac{1}{\sigma^{2}+\sigma_{i}^{2}}\right)+\sum_{i}\log\left(\sigma^{2}+\sigma_{i}^{2}\right)\\ +\sum_{i}v_{i}\log\sigma_{i}^{2}]\\ =\underset{y_{0},y_{1},\ldots ,y_{p}}{\min}[\left(n-1\right)\log\left(\sum_{i}\frac{{\left(x_{i}-\bar{\mu }\right)}^{2}}{y_{0}+y_{i}}+\sum_{i}\frac{v_{i}s_{i}^{2}}{y_{i}}\right)\\ +\log\left(\sum_{i}\frac{1}{y_{0}+y_{1}}\right)+\sum_{i}\log\left(y_{0}+y_{i}\right)+\sum_{i}v_{i}\log y_{i}]\\ +n-1-\left(n-1\right)\log\left(n-1\right). \end{array}$$

The minimizing value of *λ* is $\lambda_{0}=\left({\sum_{i}\left(x_{i}-\bar{\mu }\right)}^{2}/\left(y_{0}+y_{i}\right)+\sum_{i}v_{i}s_{i}^{2}/y_{i}\right)/\left(n-1\right)$, and the objective function in (57) is homogeneous of degree 0 in *y*’s. If *y*_0_, *y*_1_, …, *y_p_* is a solution to the problem (57), then ${\tilde{\sigma }}^{2}=\lambda_{0}y_{0}$, ${\tilde{\sigma }}_{1}^{2}=\lambda_{0}y_{1}$, …, ${\tilde{\sigma }}_{p}^{2}=\lambda_{0}y_{p}$ is a REML solution.

When *y*_0_ > 0, the function in (57) takes the form

(58) $$\begin{array}{c} \left(n-1\right)\log\left(\sum_{i}\frac{{\left(x_{i}-\bar{\mu }\right)}^{2}}{y_{0}+y_{i}}+\sum_{i}\frac{v_{i}s_{i}^{2}}{y_{i}}\right)\\ +\log\left(\sum_{i}\frac{1}{y_{0}+y_{i}}\right)-\sum_{i}\log\left(\frac{1}{y_{0}+y_{i}}\right)+\sum_{i}v_{i}\log y_{i}. \end{array}$$

Its derivative with respect to *y_i_* is

(59) $$\begin{array}{l} -\left(n-1\right)\frac{\frac{{\left(x_{i}-\bar{\mu }\right)}^{2}}{{\left(y_{0}+y_{i}\right)}^{2}}+\frac{v_{i}s_{i}^{2}}{y_{i}^{2}}}{\sum_{j}\left[\frac{{\left(x_{j}-\bar{\mu }\right)}^{2}}{y_{0}+y_{j}}+\frac{v_{i}s_{j}^{2}}{y_{j}}\right]}\\ \;-\frac{\frac{1}{{\left(y_{0}+y_{i}\right)}^{2}}}{\sum_{j}\frac{1}{y_{0}+y_{j}}}+\frac{1}{y_{0}+y_{i}}+\frac{v_{i}}{y_{i}}. \end{array}$$

As in Sec. 2.2, for a fixed value *λ*_0_ = 1 we can use an iterative scheme which is based on (59). However, now one has to specify initial values of *y*^(0)^, ${\bar{\mu }}_{0}$ and of *A*^(0)^ = ∑(*y*_0_ + *y_j_*)^−1^ after which the cubic equations for *υ_i_* = *y*_0_/*y_i_*,

(60) $$\begin{array}{l} \upsilon_{i}{\left(1+\upsilon_{i}\right)}^{2}v_{i}s_{i}^{2}-{\left(1+\upsilon_{i}\right)}^{2}v_{i}y_{0}-\left(1+\upsilon_{i}\right)y_{0}\\ \;+\upsilon_{i}/A+\upsilon_{i}{\left(x_{i}-\bar{\mu }\right)}^{2}=0 \end{array}$$

are solved for *i* = 1, …, *p*, with updating as in (47) and (48). Each of these equations has either one or three positive roots. If there is just one root, then it defines *v_i_*, out of three roots the largest is taken.

If *y* = 0, then with the vectors *e* = (1, …, 1)*^T^*, *z* = (*z*_1_,…, (*z_p_*)*^T^*, $z_{i}=1/\sigma_{i}^{2}$, and *p* × *p* symmetric matrix ℝ defined by positive elements $\left({\left(x_{i}-x_{j}\right)}^{2}+v_{i}s_{i}^{2}+v_{j}s_{j}^{2}\right)/2$, (58) takes the form

(61) $$\underset{z_{1},\ldots ,z_{p}>0}{\min}\left[\left(n-1\right)\log\left(z^{T}\mathbb{R}z\right)-\left(n-2\right)\log\left(z^{T}e\right)-\sum_{i}n_{i}\log\;z_{i}\right].$$

The gradient of the function in (61)

(62) $$\frac{2\left(n-1\right)\mathbb{R}z}{z^{T}\mathbb{R}z}-\left(n-2\right)\frac{e}{z^{T}e}-\frac{\vec{n}}{z},$$

and its Hessian is

(63) $$\begin{array}{l} \nabla^{2}F=\frac{2\left(n-1\right)\mathbb{R}}{z^{T}\mathbb{R}z}-\frac{4\left(n-1\right)\mathbb{R}z^{T}z\mathbb{R}}{{\left(z^{T}\mathbb{R}z\right)}^{2}}+\frac{\left(n-2\right)ee^{T}}{{\left(z^{T}e\right)}^{2}}\\ \;+diag\left(\frac{\vec{n}}{z^{2}}\right). \end{array}$$

where $\vec{n}/z$ is the vector with coordinates *n_i_* / *z_i_*, *i* = 1, …, *p*, leads to the iteration scheme,

(64) $$\begin{array}{c} \frac{\vec{n}}{z^{\left(k+1\right)}}\propto \frac{2\left(n-1\right)\mathbb{R}z^{\left(k\right)}}{{\left(z^{\left(k\right)}\right)}^{T}\mathbb{R}z^{\left(k\right)}}-(n-2)e,\\ z_{i}^{\left(0\right)}=\frac{s_{i}^{-2}}{\sum_{k}s_{k}^{-2}},\;e^{T}\;z^{\left(k\right)}=1, \end{array}$$

*k* = 0, 1, …, which converges fast.

The solutions obtained via (60) and (64) are to be compared by evaluating *RL* in (57).

### 3.2 REML *p* = 2

To find the REML solutions ${\tilde{\sigma }}_{i}^{2}$ and ${\tilde{\sigma }}^{2}$ when *p* = 2 notice that (51) takes the form

(65) $$\begin{array}{c} RL=\left(\frac{{\left(x_{1}-x_{2}\right)}^{2}}{2y+\sigma_{1}^{2}+\sigma_{2}^{2}}+\log\left(2y+\sigma_{1}^{2}+\sigma_{2}^{2}\right)\right)\\ +v_{1}\left(\frac{s_{1}^{2}}{\sigma_{1}^{2}}+\log\sigma_{1}^{2}\right)+v_{2}\left(\frac{s_{2}^{2}}{\sigma_{2}^{2}}+\log\sigma_{2}^{2}\right), \end{array}$$

which shows that if ${\left(x_{1}-x_{2}\right)}^{2}\geq s_{1}^{2}+s_{2}^{2}$,

(66) $${\tilde{\sigma }}_{i}^{2}=s_{i}^{2},i=1,2,$$

(67) $${\tilde{\sigma }}^{2}=\frac{1}{2}\left[{\left(x_{1}-x_{2}\right)}^{2}-s_{1}^{2}-s_{2}^{2}\right].$$

Indeed this choice simultaneously minimizes each of three bracketed terms in (65) guaranteeing the global minimum at this point. When ${\left(x_{1}-x_{2}\right)}^{2}<s_{1}^{2}+s_{2}^{2}$, as we will see, *σ*^2^ = 0. To find $\sigma_{i}^{2}$, *i* = 1, 2, one can employ the Groebner basis of the polynomial equations (56) which in the notation of Sec. 2.1 takes the form

(68) $$u^{2}\left(u+\upsilon -1\right)+\left(v_{1}u-z_{1}\right){\left(u+\upsilon \right)}^{2}=0$$

(69) $$v^{2}\left(u+\upsilon -1\right)+\left(v_{2}\upsilon -z_{2}\right){\left(u+\upsilon \right)}^{2}=0.$$

This Groebner basis consists of three polynomials

(70) $$G_{3}\left(u,\upsilon \right)=a_{0}u^{2}+a_{1}\upsilon^{5}+a_{2}\upsilon^{4}+a_{3}\upsilon^{3}+a_{4}\upsilon^{2},$$

whose coefficients are not needed here,

(71) $$\begin{array}{l} G_{4}\left(u,v\right)=b_{0}u\upsilon +b_{1}\upsilon^{5}+b_{2}\upsilon^{4}+b_{3}\upsilon^{3}+b_{4}\upsilon^{2},\\ b_{0}=-2n_{1}z_{2}^{2}\left[n_{1}z_{1}z_{2}+2\left(n_{2}-1\right)z_{1}+\left(n_{1}-2\right)z_{2}+n_{1}-2\right],\\ b_{1}=-n_{2}\left(n-1\right)\left(n-2\right){\left(n_{1}+2n_{2}-2\right)}^{2},\\ b_{2}=-{\left(n_{1}+2n_{2}-2\right)}^{2}\left(2n_{1}n_{2}+2n_{2}^{2}-n_{1}-4n_{2}+2\right)z_{1}\\ +\left(n_{1}+2n_{2}-2\right)(n_{1}^{3}+4n_{1}^{2}n_{2}+7n_{1}n_{2}^{2}+4n_{2}^{3}-5n_{1}^{2}\\ -14n_{1}n_{2}-12n_{2}^{2}+8n_{1}+12n_{2}-4)z_{2}\\ +{\left(n_{1}+2n_{2}-2\right)}^{2}\left(n_{1}^{2}+2n_{1}n_{2}+2n_{2}^{2}-3n_{1}-4n_{2}+2\right),\\ b_{3}=-{\left(n_{1}+2n_{2}-2\right)}^{2}v_{2}z_{1}^{2}\\ +2\left(n_{1}+2n_{2}-2\right)\left(n_{1}^{2}+2n_{1}n_{2}+2n_{2}^{2}-3n_{1}-4n_{2}+2\right)z_{1}z_{2}\\ -(3n_{1}^{2}n_{2}+6n_{1}n_{2}^{2}+4n_{2}^{3}-n_{1}^{2}-12n_{1}n_{2}-12n_{2}^{2}+4n_{1}\\ -12n_{2}-4)z_{2}^{2}\\ -2v_{2}{\left(n_{1}+2n_{2}-2\right)}^{2}z_{1}-2\left(n_{1}+2n_{2}-2\right)\\ \left(2n_{1}n_{2}+2n_{2}^{2}-n_{1}-4n_{2}+2\right)z_{2}\\ -2v_{2}{\left(n_{1}+2n_{2}-2\right)}^{2},\\ b_{4}=n_{1}z_{2}\left(1+z_{1}+z_{2}\right)[\left(n_{1}+2n_{2}-2\right)z_{1}+\left(n_{1}-2\right)z_{2}\\ +n_{1}+2n_{2}-2], \end{array}$$

and

(72) $$G_{5}\left(u,\upsilon \right)=c_{0}\upsilon^{6}+c_{1}\upsilon^{5}+c_{2}\upsilon^{4}+c_{3}\upsilon^{3},$$

with

$$\begin{array}{l} c_{0}=n_{2}\left(n-1\right)\left(n-2\right),\\ c_{1}=\left(2n_{2}^{2}+2n_{1}n_{2}-n_{1}-4n_{2}+2\right)z_{1}\\ -\left(n_{1}^{2}+3n_{2}^{2}+4n_{1}n_{2}-3n_{1}-6n_{2}+2\right)z_{2}\\ -n_{1}^{2}-2n_{2}^{2}-2n_{1}n_{2}+3n_{1}+4n_{2}-2,\\ c_{2}=v_{2}z_{1}^{2}-\left(2n_{1}+4n_{2}-4\right)z_{1}z_{2}\\ +\left(2n_{1}+3n_{2}-3\right)z_{2}^{2}+2v_{2}z_{1}+\left(2n_{1}+4n_{2}-4\right)z_{2}+v_{2},\\ c_{3}=-z_{2}\left[{\left(z_{1}-z_{2}\right)}^{2}+2\left(z_{1}+z_{2}\right)+1\right]. \end{array}$$

All stationary points of the restricted likelihood equations can be found by solving the cubic equation *G*_5_(*υ*) = 0 for *υ* > 0, and substituting this solution in (71), allowing one to express *u* through *v*,

$$u=-\upsilon \left(b_{1}\upsilon^{3}+b_{2}\upsilon^{2}+b_{3}\upsilon +b_{4}\right)/b_{0}.$$

Thus, in this case there are 3 complex non-zero roots pairs *u*, *v*.

Another approach is to use the argument in Sec. 3.1 which for a fixed sum 2*y*_0_ + *y*_1_ + *y*_2_ = 1, leads to the optimization problem,

(73) $$\underset{\begin{array}{l} y_{1}+y_{2}\leq 1\\ y_{1}>0,y_{2}>0 \end{array}}{\min}\left[\left(n-1\right)\log\left(1+\frac{z_{1}}{y_{1}}+\frac{z_{2}}{y_{2}}\right)+\sum v_{i}\log y_{i}\right],$$

If ${\left(x_{1}-x_{2}\right)}^{2}<s_{1}^{2}+s_{2}^{2}$, then according to Lemma 1 in the Appendix, for a fixed sum *y*_1_ + *y*_2_ = *y*, the minimum in (73) monotonically decreases when 0 < *y* ≤ 1, so that this minimum is attained at the boundary, *y*_1_ + *y*_2_ = 1, in which case indeed ${\tilde{\sigma }}^{2}=0$. Thus, one can solve the cubic equation for *w* = 1 − 2*y*_1_,

(74) $$\begin{array}{l} \left(n-2\right)w^{3}-\left(4\Delta +2\delta \right)w^{2}-\left(n+8\Delta \delta -4n\bar{z}-2\right)w\\ \;-4\Delta \left(n-1\right)-2\delta \left(1+4\bar{z}\right)=0, \end{array}$$

where *δ* = (*n*_2_ − *n*_1_)/2, and as before $\bar{z}=\left(z_{1}+z_{2}\right)/2$, Δ = (*z*_2_ − *z*_1_)/2.

An alternative method is to use the iteration scheme (64) to solve (57).

## 4. Known Variances

### 4.1 Conditions and Bounds for Strictly Positive Variance Estimates

In view of the rather complicated nature of likelihood equations, in many situations it is desirable to have a simpler estimating method for the common mean *μ*. The most straightforward way is to assume that the variances $\sigma_{i}^{2}$, *i* = 1, …, *p*, are known. In this case, essentially suggested in Ref. [15], but also pursued in Refs. [16, 17], these variances are taken to be $s_{i}^{2}$ (or a multiple thereof). Because of (3), the only parameter to estimate is *y* = *σ*^2^.

We give here upper bounds on the ML estimator ${\hat{\sigma }}^{2}$ and on the REML estimator ${\tilde{\sigma }}^{2}$.

**Theorem 4.1** Assume that the variances $\sigma_{i}^{2}$, *i* = 1, …, *p*, *are known. Then*

(75) $${\hat{\sigma }}^{2}\leq \frac{1}{2}\underset{i\neq j}{\max}{\left[\frac{{\left(p-1\right)}^{2}{\left(x_{i}-x_{j}\right)}^{2}}{p}-\left(\sigma_{i}^{2}+\sigma_{j}^{2}\right)\right]}_{+},$$

*and*

(76) $${\tilde{\sigma }}^{2}\leq \frac{1}{2}\underset{i\neq j}{\max}{\left[\left(p-1\right){\left(x_{i}-x_{j}\right)}^{2}-\left(\sigma_{i}^{2}+\sigma_{j}^{2}\right)\right]}_{+}.$$

*In particular, if*

(77) $$\underset{1\leq i<j\leq p}{\max}\frac{{\left(x_{i}-x_{j}\right)}^{2}}{\sigma_{i}^{2}+\sigma_{j}^{2}}\leq \frac{p}{{\left(p-1\right)}^{2}},$$

*then* ${\hat{\sigma }}^{2}=0$. *If*

(78) $$\underset{1\leq i<j\leq p}{\max}\frac{{\left(x_{i}-x_{j}\right)}^{2}}{\sigma_{i}^{2}+\sigma_{j}^{2}}\leq \frac{1}{p-1},$$

${\tilde{\sigma }}^{2}=0$.

*Proof*: We prove first that

(79) $$|QP^{\prime \prime }-Q^{\prime }P^{\prime }|\leq \left(p-1\right)\underset{1\leq i<j\leq p}{\max}\frac{{\left(x_{i}-x_{j}\right)}^{2}}{\sigma_{i}^{2}+\sigma_{j}^{2}}P^{\prime }P^{\prime \prime }.$$

Indeed, one can write

(80) $$QP^{\prime \prime }-Q^{\prime }P^{\prime }=\sum_{k,\ell }k\left(k-1-\ell \right)q_{p-2-\ell }S_{p-k}y^{k+\ell -2},$$

and Lemma 3 in the Appendix shows that since (*p* − 1 − *ℓ*)| *k* − 1 − *ℓ* | ≤ (*p* − 1)(*k* − 1), (79) holds.

One gets for the polynomial *H* in (53),

(81) $$H\geq P^{\prime }P^{\prime \prime }\left[1-\left(p-1\right)\underset{1\leq i<j\leq p}{\max}\frac{{\left(x_{i}-x_{j}\right)}^{2}}{\sigma_{i}^{2}+\sigma_{j}^{2}}\right],$$

which is positive under condition (78). Then ${\tilde{\sigma }}^{2}=0$.

Similarly (77) and (54) imply that

(82) $$F\geq P^{\prime }\left[{\left(P^{\prime }\right)}^{2}-\left(p-1\right)\underset{1\leq i<j\leq p}{\max}\frac{{\left(x_{i}-x_{j}\right)}^{2}}{\sigma_{i}^{2}+\sigma_{j}^{2}}PP^{\prime \prime }\right]\geq 0.$$

To prove (76) observe that with *y* replaced by *y* + *a* a formula like (79) holds for any positive *a*. The only modification is that $S_{p-\ell }\left(\sigma_{1}^{2},\ldots ,\sigma_{p}^{2}\right)$ is replaced by $S_{p-\ell }\left(a+\sigma_{1}^{2},\ldots ,a+\sigma_{p}^{2}\right)$ and the *H*^(^*^k^*^)^*(a) / k*! represent polynomial coefficients. Then a version of Lemma 3 in the Appendix states that

(83) $$\begin{array}{l} \frac{Q^{\left(\ell \right)}\left(a\right)}{\ell !}\leq \left(\ell +1\right)\left(p-\ell -1\right)\\ \underset{1\leq i<j\leq p}{\max}\left[\frac{{\left(x_{i}-x_{j}\right)}^{2}}{2a+\sigma_{i}^{2}+\sigma_{j}^{2}}\right]\frac{P^{\left(\ell +1\right)}\left(a\right)}{\left(\ell +1\right)!}\\ =\left(p-\ell -1\right)\underset{1\leq i<j\leq p}{\max}\left[\frac{{\left(x_{i}-x_{j}\right)}^{2}}{2a+\sigma_{i}^{2}+\sigma_{j}^{2}}\right]\frac{P^{\left(\ell +1\right)}\left(a\right)}{\ell !}. \end{array}$$

The argument, as before, shows now that if $\max_{1\leq i<j\leq p}{\left(x_{i}-x_{j}\right)}^{2}/\left(2a+\sigma_{i}^{2}+\sigma_{j}^{2}\right)\leq {\left(p-1\right)}^{-1}$, then *H*^(^*^k^*^)^ (*a*) ≥ 0, *k* = 0, …, 2 *p* −3. It follows that ${\tilde{\sigma }}^{2}\leq a$, so that (76) is valid with $a=2^{-1}\max{\left[\left(p-1\right){\left(x_{i}-x_{j}\right)}^{2}-\left(\sigma_{i}^{2}+\sigma_{j}^{2}\right)\right]}_{+}$. The proof of (75) is similar. _+_

When *p* = 2, the bounds (75) and (76) are sharp as (24) and (67) show. When *p* increases, their accuracy decreases. Section 4.2 gives necessary and sufficient conditions for ${\tilde{\sigma }}^{2}=0$ when *p* = 3. It is possible to get better estimates under additional conditions. For example, if the ordering of sequences $S_{p-\ell }\left({\bar{\sigma }}_{ij}^{2}\right)\left(\sigma_{i}^{2}+\sigma_{j}^{2}\right)$ (notation of Lemma 2) and ${\left(x_{i}-x_{j}\right)}^{2}{\left(\sigma_{i}^{2}+\sigma_{j}^{2}\right)}^{-1}$ is the same, then the maximum of ${\left(x_{i}-x_{j}\right)}^{2}\left(\sigma_{i}^{2}+\sigma_{j}^{2}\right)$, 1 ≤ *i* < *j* ≤ *p*, in (77) and (78) can be replaced by their average.

### 4.2 Example: Restricted Maximum Likelihood for *p* = 3

When *p* = 3, *Q*(*y*) = *q*_0_*y* + *q*_1_, is a polynomial of degree one, and

(84) $$\begin{array}{l} H\left(y\right)=18y^{3}+3\left(6S_{1}-q_{0}\right)y^{2}\\ \;+\left(6S_{2}+4S_{1}^{2}-6q_{1}\right)y+2S_{2}S_{1}+q_{0}S_{2}-2q_{1}S_{1}\\ \;=\sum_{0}^{3}h_{3-k}y^{k} \end{array}$$

is a polynomial of degree three. If *H*(0) = *h*_3_ < 0, it has a positive root, which means that ${\tilde{\sigma }}^{2}>0$. Otherwise the existence of a positive root is related to the sign of the discriminant

(85) $$\begin{array}{l} D=h_{1}^{2}h_{2}^{2}-4h_{0}h_{2}^{3}-4h_{1}^{3}h_{3}\\ \;-27h_{0}^{2}h_{3}^{2}+18h_{0}h_{1}h_{2}h_{3}. \end{array}$$

If *D* < 0 and *h*_3_ ≥ 0, there is just one real root which must be negative, and then ${\tilde{\sigma }}^{2}=0$. If *D* ≥ 0, then there are three real roots. The condition *h*_3_ ≥ 0 implies that either two of them are positive and one is negative (so that at least one of the coefficients *h*_1_ or *h*_2_ is negative) and ${\tilde{\sigma }}^{2}>0$, or all three roots are negative (so that ${\tilde{\sigma }}^{2}>0$.)

Separation between these cases depends on the ratio $\lambda =q_{1}/q_{0}^{2}$. Let $E_{1}=S_{1}/q_{0}$, $E_{2}=S_{2}/q_{0}^{2}$. Then $E_{2}\leq E_{1}^{2}/3$, *h*_0_ = 18, *h*_1_/*q*_0_ = 18(*E*_1_−1/6), $h_{2}/q_{0}^{2}=6\left(E_{2}+2E_{1}^{2}/3-\lambda \right)$, $h_{3}/q_{0}^{3}=2E_{1}E_{2}+E_{2}-2\lambda E_{1}$. Actually, as follows from the proof of Theorem 4.1, *E*_1_ ≥ λ, but we do not use this fact here.

Algebra shows that when $E_{2}=E_{1}^{2}/3$, one has *D*= 544(*E*_1_ − 3*λ*)^2^(3*λ* +1/16 − *E*_1_), when *h_3_* = 0, i.e., if *E_2_ = λE_1_/(E*_1_ + 0.5), then $D=36{\left(4E_{1}^{3}+E_{1}^{2}-3\lambda \right)}^{2}\left(8E_{1}^{3}-20E_{1}^{2}-10E_{1}+1+48\lambda \right){\left(1+2E_{1}\right)}^{-3}$, and on the curve *h*_2_ = 0, i.e., when $E_{2}=\lambda -2E_{1}^{2}/3$, *D* factorizes as follows, $D=36\left(4E_{1}^{3}+2E_{1}^{2}-3\lambda \right)\left(108E_{1}^{3}-162E_{1}^{2}-18E_{1}+81\lambda \right)$. Let ${\hat{E}}_{1}$ be the smallest positive root of the cubic equation

(86) $$8E_{1}^{3}-20E_{1}^{2}-10E_{1}+1+48\lambda =0,$$

which is defined for *λ* < 0.5137…, and let ${\overset{⌣}{E}}_{1}$ be the (only) positive root of the equation

(87) $$4E_{1}^{3}+2E_{1}^{2}-3\lambda =0.$$

The discriminant of (87) is negative, and according to Descartes’s rule there are always two complex roots, and one positive root. It is easy to check that ${\overset{⌣}{E}}_{1}={\overset{⌣}{E}}_{1}(\lambda )$ is monotonically increasing in *λ* < 0 and ${\overset{⌣}{E}}_{1}(1/16)=1/4$.

**Fig. 5:**
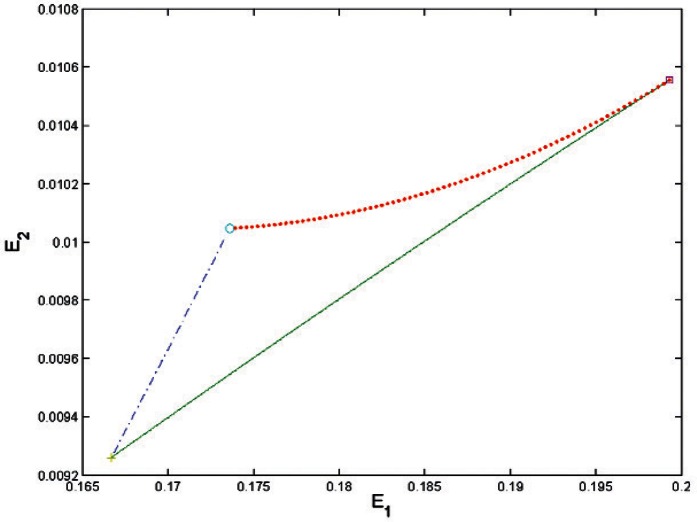
The region where *h*_3_ ≥ 0 and ${\tilde{\sigma }}^{2}>0$ when *λ* = 1/27. The solid line is *h*_3_ ≥ 0, the dash-dotted line is $E_{2}=E_{1}^{2}/3$, and the dotted line is *D* = 0. The point *M*_1_ is marked by +, the point *M*_2_ is marked by a square, the point *M*_3_ by *o*.

In this notation, when *λ ≤* 1/16 (so that *h_2_ ≤* 0 implies that *h_1_ ≤* 0), the region $\left\{(E_{1},E_{2}):E_{2}\leq E_{1}^{2}/3,h_{3}\geq 0,\sigma_{2}>0\right\}$, is formed by three curves: (*i*) *h_3_* = 0 between the point $M_{1}=((\sqrt{1+48\lambda }-1)/4$, $\left(1+24\lambda -\sqrt{1+48\lambda )}/24\right)$, where *h_3_* = 0 intersects the curve $E_{2}=E_{1}^{2}/3$, and the point $\left({\overset{⌣}{E}}_{1},\lambda -2{\overset{⌣}{E}}_{1}^{2}/3\right)$ on *h_2_ = 0, (ii)* $E_{2}=E_{1}^{2}/3$ between *M_1_* and the point $M_{2}=(E_{1}=3\lambda +1/6,E_{1}^{2}/3)$ where *D* = 0 intersects $E_{2}=E_{1}^{2}/3$, and by *(i i i)* the cubic in *E_2_* curve corresponding to the equation *D* = 0, which connects the point *M_2_* and the point $M_{3}=(3\lambda +1/6,E_{1}^{2}/3).$. See Fig. 5. If *λ* <2/81, this region also includes the part of the curve *h_3_* = 0 where ${\overset{⌣}{E}}_{1}\leq {\hat{E}}_{1}$, but the probability of having the likelihood solution there is zero.

When *λ* > 1/16, one has $E_{1}>1/4={\overset{⌣}{E}}_{1}(1/16)$, so that *h_1_* >0. Also then*E*_2_ *>λ/3*, ${\overset{⌣}{E}}_{1}>\sqrt{\lambda }$, so that *h*_2_ >0. Thus in this situation ${\tilde{\sigma }}^{2}>0$ if and only if *h*_3_ ≥0.

## 5. Moment-Type Equations

### 5.1 Weighted Means Statistics

When the within-lab and between-lab variances $\sigma_{i}^{2}$ and *σ*^2^ are known, the best (in terms of the mean squared error) unbiased estimator of the treatment effect *μ* in the model (1) is a weighted means statistic (3) with $w_{i}=w_{i}^{0}=1/(\sigma^{2}+\sigma_{i}^{2}).$ Even without the normality assumption for these optimal weights, $E\sum_{i}w_{i}^{0}{\left(x_{i}-\bar{\mu }\right)}^{2}=p-1$, and

(88) $$E{\left(\bar{\mu }-\mu \right)}^{2}=\frac{1}{\sum_{i}w_{i}^{0}}=\frac{1}{\sum_{i}{\left(\sigma^{2}+\sigma_{i}^{2}\right)}^{-1}}.$$

If $Var(x_{i})=\sigma_{i}^{2}+\sigma^{2}$, but the weights *w_i_* are arbitrary,

(89) $$\begin{array}{c} E\sum_{i}w_{i}{\left(x_{i}-\bar{\mu }\right)}^{2}=\sum_{1}^{p}(\sigma^{2}+\sigma_{i}^{2})w_{i}-\frac{\sum_{1}^{p}(\sigma^{2}+\sigma_{i}^{2})w_{i}^{2}}{\sum_{1}^{p}w_{i}}\\ =\sigma^{2}\left[\sum_{1}^{p}w_{i}-\frac{\sum_{1}^{p}w_{i}^{2}}{\sum_{1}^{p}w_{i}}\right]+\sum_{1}^{p}\sigma_{i}^{2}w_{i}-\frac{\sum_{1}^{p}\sigma_{i}^{2}w_{i}^{2}}{\sum_{1}^{p}w_{i}}. \end{array}$$

In particular, when $w_{i}=1/\sigma_{i}^{2}$

(90) $$E\sum_{i}\frac{{\left(x_{i}-\bar{\mu }\right)}^{2}}{\sigma_{i}^{2}}=p-1+\sigma^{2}\left[\sum_{1}^{p}\frac{1}{\sigma_{i}^{2}}-\frac{\sum_{1}^{p}\frac{1}{\sigma_{i}^{4}}}{\sum_{1}^{p}\frac{1}{\sigma_{i}^{2}}}\right].$$

The simplest estimate of the within-trials variances $\sigma_{i}^{2}$ is by the available $s_{i}^{2}$, but the problem of estimating the between-trial component of variance σ^2^ remains.

### 5.2 DerSimonian-Laird Procedure

By employing the idea behind the method of moments, DerSimonian and Laird [18] made use of the identity (90) as an estimating equation for *μ* and *σ*^2^, provided that $\sigma_{i}^{2}$ are estimated by $s_{i}^{2}$, in the following way. For weights of the form

(91) $$w_{i}=\frac{1}{y+s_{i}^{2}},$$

determine a non-negative *y* = *y*_DL_ from the formula,

(92) $$y_{DL}=\max\left[0,\frac{\sum_{i}s_{i}^{-2}{\left(x_{i}-{\tilde{x}}^{0}\right)}^{2}-p+1}{\sum_{i=1}^{p}s_{i}^{-2}-\sum_{i=1}^{p}s_{i}^{-4}{\left(\sum_{i=1}^{p}s_{i}^{-2}\right)}^{-1}}\right]$$

and put ${\tilde{x}}_{DL}=\left(\sum_{i=1}^{p}x_{i}{\left(y_{DL}+s_{i}^{2}\right)}^{-2}\right)/\sum_{i=1}^{p}{\left(y_{DL}+s_{i}^{2}\right)}^{-2}$. Here ${\tilde{x}}^{0}=\left(\sum_{i=1}^{p}x_{i}s_{i}^{-2}\right)/\sum_{i=1}^{p}s_{i}^{-2}$, is one of traditional estimators for the common mean (the Graybill-Deal estimator). In other words, the statistic ${\tilde{x}}^{0}$ and the weights $w_{i}=s_{i}^{-2}$ corresponding to *σ*^2^ = 0, are used to evaluate the sum $\sum_{i}s_{i}^{-2}{\left(x_{i}-{\tilde{x}}^{0}\right)}^{2}$ which is then employed to estimate *σ*^2^ via (90).

### 5.3 Mandel-Paule Method

The Mandel-Paule algorithm uses weights of the form (91) as well, but now *y* = *y_MP_*, which is designed to approximate *σ*^2^, is found from the moment-type estimating equation,

(93) $$\sum_{i=1}^{p}\frac{{\left(x_{i}-\tilde{x}\right)}^{2}}{y_{MP}+s_{i}^{2}}=p-1.$$

See Refs. [15], [19]. The *modified Mandel-Paule* procedure with *y* = *y_MMP_* is defined as above, but *p* − 1 in the right-hand side of (93) is replaced by *p*, i.e.,

(94) $$\sum_{i=1}^{p}\frac{{\left(x_{i}-\tilde{x}\right)}^{2}}{y_{MP}+s_{i}^{2}}=p.$$

Notice that when *p* = 2, the DerSimonian-Laird estimator coincides with the Mandel-Paule rule, as, in this case,

(95) $$y_{MP}=y_{DL}=\frac{1}{2}\max\left[0,{\left(x_{1}-x_{2}\right)}^{2}-s_{1}^{2}-s_{2}^{2}\right],$$

so that this estimator is similar to the REML estimates estimates (66) and (67). In the general case, both of these rules set *y* = 0, when

(96) $$\sum_{i=1}^{p}\frac{{\left(x_{i}-\tilde{x}\right)}^{2}}{s_{i}^{2}}\leq p-1.$$

It was shown in Ref. [20] that the modified Mandel-Paule is characterized by the following fact: the ML estimator ${\hat{\sigma }}^{2}$ of *σ*^2^ coincides with *y_MMP_*, if in the reparametrized version of the likelihood equation the weights *w_i_* admit representation (91). As a consequence, the corresponding weighted means statistic (3) must be close to the ML estimator, so that the modified Mandel-Paule estimator approximates its ML counterpart. Thus, the modified Mandel-Paule estimator can be interpreted as a procedure which uses weights of the form $1/\left(y+s_{i}^{2}\right)$, instead of solutions of the likelihood equation that are more difficult to find, and still maintains the same estimate of *σ*^2^ as ML.

A similar interpretation holds for the original Mandel-Paule rule and the REML function [21]. For this reason both Mandel-Paule rules are quite natural. It is also suggestive to use the weights (91) with *y* = *y_MP_* determined from the Mandel-Paule equation (93) as a first approximation when solving the likelihood equations via the iterative scheme (47) and (48).

### 5.4 Uncertainty Assessment

It is tempting to use formula (88) to obtain an estimator of the variance of $\tilde{x}$. For example, DerSimonian and Laird [18] suggested an approximate formula for the estimate of the variance of their estimator,

(97) $$\hat{Va}r\left({\tilde{x}}_{DL}\right)=\frac{1}{\sum_{i}{\left(y_{DL}+s_{i}^{2}\right)}^{-1}}.$$

Similarly motivated by (88), Paule and Mandel [19] suggested to use ${\left[\sum_{i}{\left(y_{MP}+s_{i}^{2}\right)}^{-1}\right]}^{-1}$ to estimate the variance of ${\tilde{x}}_{MP}$. However these estimators typically underestimate the true variance, Ref. [5]. They are not GUM consistent [22] in the sense that the variance estimate is not representable as a quadratic form in deviations $x_{i}-\tilde{x}$.

Horn, Horn and Duncan [23] in the more general context of linear models have suggested the following GUM consistent estimate of *Var* $\left(\tilde{x}\right)$, which has the form $\sum_{i}\omega_{i}^{2}{\left(x_{i}-\tilde{x}\right)}^{2}/\left(1-\omega_{i}\right)$, with ω*_i_* = *w_i_*/∑*_k_w_k_*. When the plug-in weights are $\omega_{i}={\left(y+s_{i}^{2}\right)}^{-1}/\sum_{k}{\left(y+s_{k}^{2}\right)}^{-1}$, *i* = 1, …, *p*, this estimate is

(98) $$\delta =\frac{\sum_{i}\frac{{\left(x_{i}-\tilde{x}\right)}^{2}}{{\left(y+s_{i}^{2}\right)}^{2}}{\left[\sum_{k:k\neq i}\frac{1}{y+s_{k}^{2}}\right]}^{-1}}{\sum_{i}\frac{1}{y+s_{i}^{2}}}.$$

Simulations show that (98) gives good confidence intervals of the form, $\tilde{x}\pm t_{\alpha /2}(p-1)\sqrt{\delta_{2}}$. For the Mandel-Paule rule or the DerSimonian-Laird procedure they outperform the intervals, $\tilde{x}\pm t_{\alpha /2}(p-1)\sqrt{\hat{Va}r\left({\tilde{x}}_{DL}\right)}$. Still when all sample means are close $x_{i}=\tilde{x}$, then *y_DL_* = 0 and the uncertainty estimate ${\left(\sum_{i}s_{i}^{-2}\right)}^{-1}$ may be a more satisfactory answer than *δ* ≈ 0.

When *p* = 2,

(99) $$\delta =\frac{{\left(x_{1}-x_{2}\right)}^{2}\left(y+s_{1}^{2}\right)\left(y+s_{2}^{2}\right)}{{\left(2y+s_{1}^{2}+s_{2}^{2}\right)}^{2}}.$$

In this case *δ* is an increasing function of *y* with the largest value (*x*_1_ − *x*_2_)^2^/4 attained when *y* → ∞.

An alternative method of obtaining confidence intervals for *μ* on the basis of REML estimators was suggested in Ref. [24] for an adjusted estimator of $\hat{Va}r(\tilde{\mu })$ based on the inverse of the Fisher information matrix. This (not GUM consistent) estimator is more complicated.

## 6. Conclusions

The original motivation for this work was an attempt to employ modern computational algebra techniques by evaluating the Groebner bases of likelihood polynomial equations for the random effects model. While this attempt leads to an explicit answer when there are two labs with no between-lab effect, it was not successful in a more general situation. The classical iterative algorithms appear to be more efficient in this application although they do not guarantee the global optimum. Simplified, method of moments based procedures, especially the DerSimonian-Laird method, deserve much wider use in interlaboratory studies.

## 7. Appendix

### 7.1 Lemma 1

**Lemma 1** *If* $\frac{z_{1}}{v_{1}}+\frac{z_{2}}{v_{2}}>1$, *then the following function* of *y*,

(100) $$\underset{y_{1}+y_{2}=y}{\min}\left[(n-1)\log\left(1+\frac{z_{1}}{y_{1}}+\frac{z_{1}}{y_{1}}\right)+\sum v_{i}\log y_{i}\right],$$

*is monotonically nondecreasing in the interval* (0, 1)

*Proof*: With *B* = (1 + *z*_1_/*y*_1_ + *z*_2_/*y*_2_) / (*n* − 1), any stationary point (*y*_1_, *y*_2_) in (100) satisfies the simultaneous equations,

(101) $$\frac{z_{1}}{y_{1}^{2}B}-\frac{v_{1}}{y_{1}}=\frac{z_{2}}{y_{2}^{2}B}-\frac{v_{1}}{y_{2}}=\zeta ,$$

where *ζ* is the Lagrange multiplier. By multiplying these equations by *y*_1_ and *y*_2_ respectively and adding them, we see that *ζ y* = 1 − 1/*B.* Notice that *B* ≤ 1 if and only if *ζ* ≤ 0, which means that *B* ≥ *z_i_*/(*v_i_y_i_*) for *i* = 1, 2. Then *z*_1_/*v*_1_ + *z*_2_/*v*_2_ ≤ 1, which contradicts the condition of the Lemma 1. Therefore, *B* > 1.

To prove Lemma 1 it suffices to show that the derivative of (100) is negative. This fact follows from the inequality

(102) $$\frac{(n-1)B^{\prime }}{B}+\frac{v_{1}{y^{\prime }}_{1}}{y_{1}}+\frac{v_{2}{y^{\prime }}_{2}}{y_{2}}=\frac{B-1}{By}<0,$$

which can be proven by differentiation of (101). Indeed for *i* = 1, 2,

(103) $$\frac{v_{i}{y^{\prime }}_{1}}{y_{i}}+\frac{z_{i}B^{\prime }}{y_{i}B^{2}}=\frac{y_{i}(B-1)}{y^{2}B}+\frac{y_{i}B^{\prime }}{yB^{2}}-\frac{2{y^{\prime }}_{i}(B-1)}{yB}.$$

Since *y*_1_ + *y*_2_ = *y*, and ${y^{\prime }}_{1}+{y^{\prime }}_{2}=1$, by adding these identities we get

(104) $$\frac{v_{1}{y^{\prime }}_{1}}{y_{1}}+\frac{v_{2}{y^{\prime }}_{2}}{y_{2}}=-\frac{B^{\prime }}{B^{2}}\left(1+\frac{z_{1}}{y_{1}}+\frac{z_{2}}{y_{2}}\right)-\frac{\left(B-1\right)}{By}.$$

### 7.2 Elementary Symmetric Polynomials: Lemmas 2 and 3

We note the following identities for elementary symmetric polynomials. For 1 ≤ *i* < *j* ≤ *p*, define ${\bar{\sigma }}_{ij}^{2}=\left(\sigma_{1}^{2},\ldots ,\sigma_{i-1}^{2},\sigma_{i+1}^{2},\ldots ,\sigma_{j-1}^{2},\sigma_{j+1}^{2},\ldots ,\sigma_{p}^{2}\right)$. Since $P(y)=\left(y^{2}+\left(\sigma_{i}^{2}+\sigma_{j}^{2}\right)y+\sigma_{i}^{2}\sigma_{j}^{2}\right)\prod_{k\neq i,j}\left(y+\sigma_{k}^{2}\right)$, one has

(105) $$S_{p-k}=S_{p-k}\left({\bar{\sigma }}_{ij}^{2}\right)+\left(\sigma_{i}^{2}+\sigma_{j}^{2}\right)S_{p-k-1}\left({\bar{\sigma }}_{ij}^{2}\right)+\sigma_{i}^{2}\sigma_{j}^{2}S_{p-k-2}\left({\bar{\sigma }}_{ij}^{2}\right).$$

Comparison of the coefficients of the polynomial $P^{\prime \prime }=\sum_{\ell =2}^{p}\ell (\ell -1)S_{p-\ell }y^{\ell -2}$ with its other expression, $2\sum_{1\leq i<j\leq p}\prod_{k\neq i,j}\left(y+\sigma_{k}^{2}\right)$, shows that

(106) $$\sum_{1\leq i<j\leq p}S_{p-\ell }\left({\bar{\sigma }}_{ij}^{2}\right)=\left(\begin{array}{c} \ell \\ 2 \end{array}\right)S_{p-\ell }.$$

By differentiating the identity, $S_{p-\ell }\left(\lambda \sigma_{1}^{2},\ldots ,\lambda \sigma_{p}^{2}\right)=\lambda^{p-\ell }S_{p-\ell }\left(\sigma_{1}^{2},\ldots ,\sigma_{p}^{2}\right)$ twice with respect to *λ*, we get

(107) $$\sum_{1\leq i<j\leq p}\sigma_{i}^{2}\sigma_{j}^{2}S_{p-2-\ell }\left({\bar{\sigma }}_{ij}^{2}\right)=\left(\begin{array}{c} p-\ell \\ 2 \end{array}\right)S_{p-\ell }.$$

Combined with (105), identities (106) and (107) demonstrate the following formula (108) (which the author was unable to find in the literature.)

**Lemma 2** *Under the notation above, for* 1 ≤ *ℓ* ≤ *p* −1,

(108) $$\sum_{1\leq i<j\leq p}\left(\sigma_{i}^{2}+\sigma_{j}^{2}\right)S_{p-1-\ell }\left({\bar{\sigma }}_{ij}^{2}\right)=\ell (p-\ell )S_{p-\ell }.$$

The following result Lemma 2 gives an upper bound on the coefficients of polynomial *Q*.

**Lemma 3** *For ℓ* = 0, 1, , …, *p* − 2,

(109) $$q_{p-2-\ell }\leq (\ell +1)(p-\ell -1)S_{p-1-\ell }\underset{1\leq i<j\leq p}{\max}\frac{{\left(x_{i}-x_{j}\right)}^{2}}{\sigma_{i}^{2}+\sigma_{j}^{2}}.$$

Indeed,

(110) $$\begin{array}{l} q_{p-2-\ell }=\sum_{1\leq i<j\leq p}{\left(x_{i}-x_{j}\right)}^{2}S_{p-2-\ell }\left({\bar{\sigma }}_{ij}^{2}\right)\\ \;=\sum_{1\leq i<j\leq p}\frac{{\left(x_{i}-x_{j}\right)}^{2}}{\sigma_{i}^{2}+\sigma_{j}^{2}}\left(\sigma_{i}^{2}+\sigma_{j}^{2}\right)S_{p-2-\ell }\left({\bar{\sigma }}_{ij}^{2}\right). \end{array}$$
